# CAR-macrophage: Breaking new ground in cellular immunotherapy

**DOI:** 10.3389/fcell.2024.1464218

**Published:** 2024-10-03

**Authors:** Ting Huang, Chenqi Bei, Zhenhua Hu, Yuanyuan Li

**Affiliations:** ^1^ Zhongshan Institute for Drug Discovery, Shanghai Institute of Materia Medica, Chinese Academy of Sciences, Zhongshan, China; ^2^ School of Chinese Materia Medica, Nanjing University of Chinese Medicine, Nanjing, China; ^3^ Shanghai Institute of Materia Medica, Chinese Academy of Sciences, Shanghai, China; ^4^ University of Chinese Academy of Sciences, Beijing, China

**Keywords:** macrophage, chimeric antigen receptor, CAR-M therapy, tumor immunotherapy, solid tumor

## Abstract

Chimeric Antigen Receptor (CAR) technology has revolutionized cellular immunotherapy, particularly with the success of CAR-T cells in treating hematologic malignancies. However, CAR-T cells have the limited efficacy of against solid tumors. To address these limitations, CAR-macrophages (CAR-Ms) leverage the innate properties of macrophages with the specificity and potency of CAR technology, offering a novel and promising approach to cancer immunotherapy. Preclinical studies have shown that CAR-Ms can effectively target and destroy tumor cells, even within challenging microenvironments, by exhibiting direct cytotoxicity and enhancing the recruitment and activation of other immune cells. Additionally, the favorable safety profile of macrophages and their persistence within solid tumors position CAR-Ms as potentially safer and more durable therapeutic options compared to CAR-T cells. This review explores recent advancements in CAR-Ms technology, including engineering strategies to optimize their anti-tumor efficacy and preclinical evidence supporting their use. We also discuss the challenges and future directions in developing CAR-Ms therapies, emphasizing their potential to revolutionize cellular immunotherapy. By harnessing the unique properties of macrophages, CAR-Ms offer a groundbreaking approach to overcoming the current limitations of CAR-T cell therapies, paving the way for more effective and sustainable cancer treatments.

## 1 Introduction

The landscape of cellular immunotherapy has been profoundly transformed by the advent of chimeric antigen receptor (CAR) technology (Biglari et al., 2006; Steinbach et al., 2022; Velasco-de Andres et al., 2023), which generates synthetic chimeric proteins that enable immune cells to recognize specific antigens on the surface of cancer cells, thereby triggering the immune cells’ ability to eliminate these cells (Kennedy and Salama, 2020; Lai et al., 2020). Particularly CAR-T cells have shown remarkable success in treating certain hematologic malignancies (Dana et al., 2021; Hong et al., 2020; Sterner and Sterner, 2021), but CAR-T cell therapies have limited efficacy against solid tumors (Halim and Maher, 2020; Kosti et al., 2018; Zhang et al., 2018; Brown and Adusumilli, 2016). As a result, there is a growing need to explore alternative immune cell types that can overcome these limitations and expand the therapeutic potential of CAR technology.

Macrophages, key players in the innate immune system (Cassetta and Pollard, 2018; Conte, 2022), are emerging as promising candidates for CAR-based therapies (DeNardo and Ruffell, 2019; Shin et al., 2023). Unlike T cells, macrophages possess inherent abilities to infiltrate solid tumors, modulate the tumor microenvironment, and sustain prolonged anti-tumor activity (Guerriero, 2018; Noy and Pollard, 2014; Sloas et al., 2021; Liu et al., 2022a). CAR-macrophages (CAR-Ms) have been engineered to combine these natural advantages with the specificity and potency of CAR technology, offering a novel approach to cancer immunotherapy (Unver, 2023). Preclinical studies have demonstrated the potential of CAR-Ms to effectively target and destroy tumor cells, even within the hostile microenvironment of solid tumors (Klichinsky et al., 2020; Chen et al., 2023). CAR-Ms not only exhibit direct cytotoxicity against cancer cells but also enhance the recruitment and activation of other immune cells (Su et al., 2022), thereby orchestrating a comprehensive anti-tumor response (Klichinsky et al., 2020). Furthermore, the safety profile of macrophages, coupled with their ability to persist and function within tumors, positions CAR-Ms as a potentially safer and more durable therapeutic option compared to CAR-T cells (Brudno and Kochenderfer, 2019). In this review, we provide a comprehensive and systematic overview of CAR-Ms, focusing on key aspects such as CAR structure, cell sources, and the progression of both preclinical and clinical studies. We also examine the challenges and future directions in the development of CAR-M therapies, highlighting their potential to revolutionize the field of cellular immunotherapy. This review aims to offer readers a concise and comprehensive overview of the rapidly evolving area of CAR-M research, helping them to grasp its current progress and future promise.

## 2 An overview of CAR-Ms

### 2.1 CAR structure and acting mechanism of CAR-Ms

**FIGURE 1:**
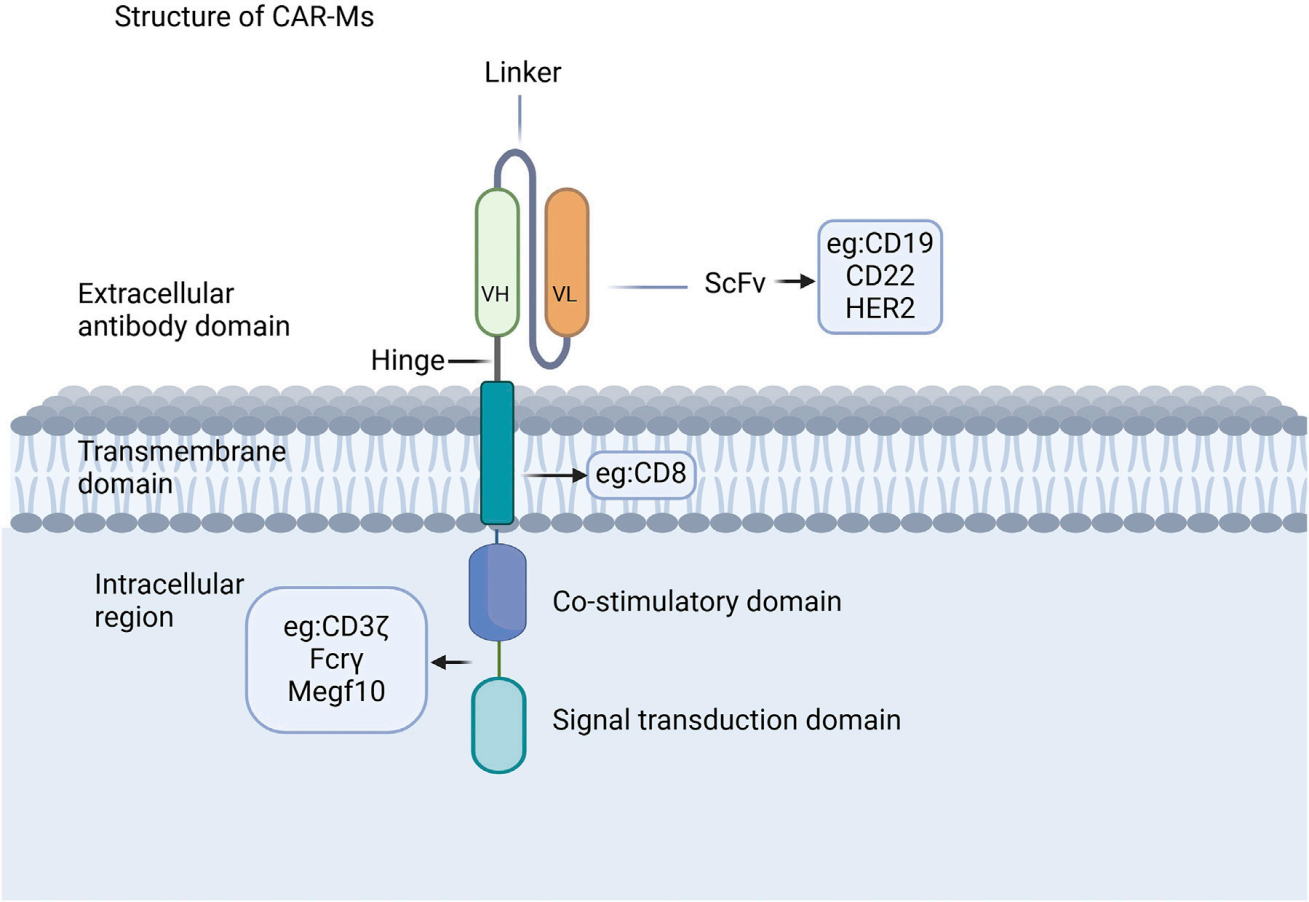
CAR structures of CAR-Ms. The CAR structure in CAR-Ms shares similarities with that of CAR-T cells, consisting of three core components: an extracellular domain, a transmembrane domain, and an intracellular domain.

The CAR structure in CAR-Ms shares similarities with that of CAR-T cells, comprising three core components: an extracellular domain, a transmembrane domain, and an intracellular domain (Figure 1) (Chen et al., 2024; Li J. et al., 2024; Li J. et al., 2022; Liu Y. et al., 2022; Wu et al., 2022; Yang et al., 2024). The extracellular domain, containing a single-chain variable fragment (scFv) that is primarily composed of the variable light chain and variable heavy chain, is responsible for recognizing antigens (Thakar et al., 2019; Zhang et al., 2022). CAR-Ms have been reported to target CD19, CD22, and HER2 on tumor cells for tumor clearance (Morrissey et al., 2018; Zhang et al., 2020; Zhang et al., 2019), and there are also reports indicating that they target the FAP protein for the treatment of liver fibrosis (Kim and Seki, 2023; Wegrzyniak et al., 2021; Yang AT. et al., 2023). The co-stimulatory and signal transduction domains are crucial for the proinflammatory and phagocytic functions of CAR-Ms. Several ligands from the immunoglobulin superfamily and the TNF receptor family, such as CD80, CD86, 4-1BBL, and OX40L, have been shown to enhance macrophage phagocytosis and cytokine secretion upon antigen binding (Stephan et al., 2007; Lei et al., 2024). CAR macrophages can utilize the same signaling domain as CAR-T cells, specifically CD3ζ, which contains the immunoreceptor tyrosine-based activation motif (ITAM) (Bettini et al., 2017). Studies on intracellular signaling pathways have shown that CAR macrophages containing CD3 are able to effectively exert their phagocytic function (Liu et al., 2022a). Additionally, a dual-signaling CAR incorporating CD3ζ and Toll-like receptor (TIR) domains has been designed to enhance target phagocytosis, promote antigen-dependent M1 polarization, and increase resistance to M2 polarization (Lei et al., 2024).

**FIGURE 2:**
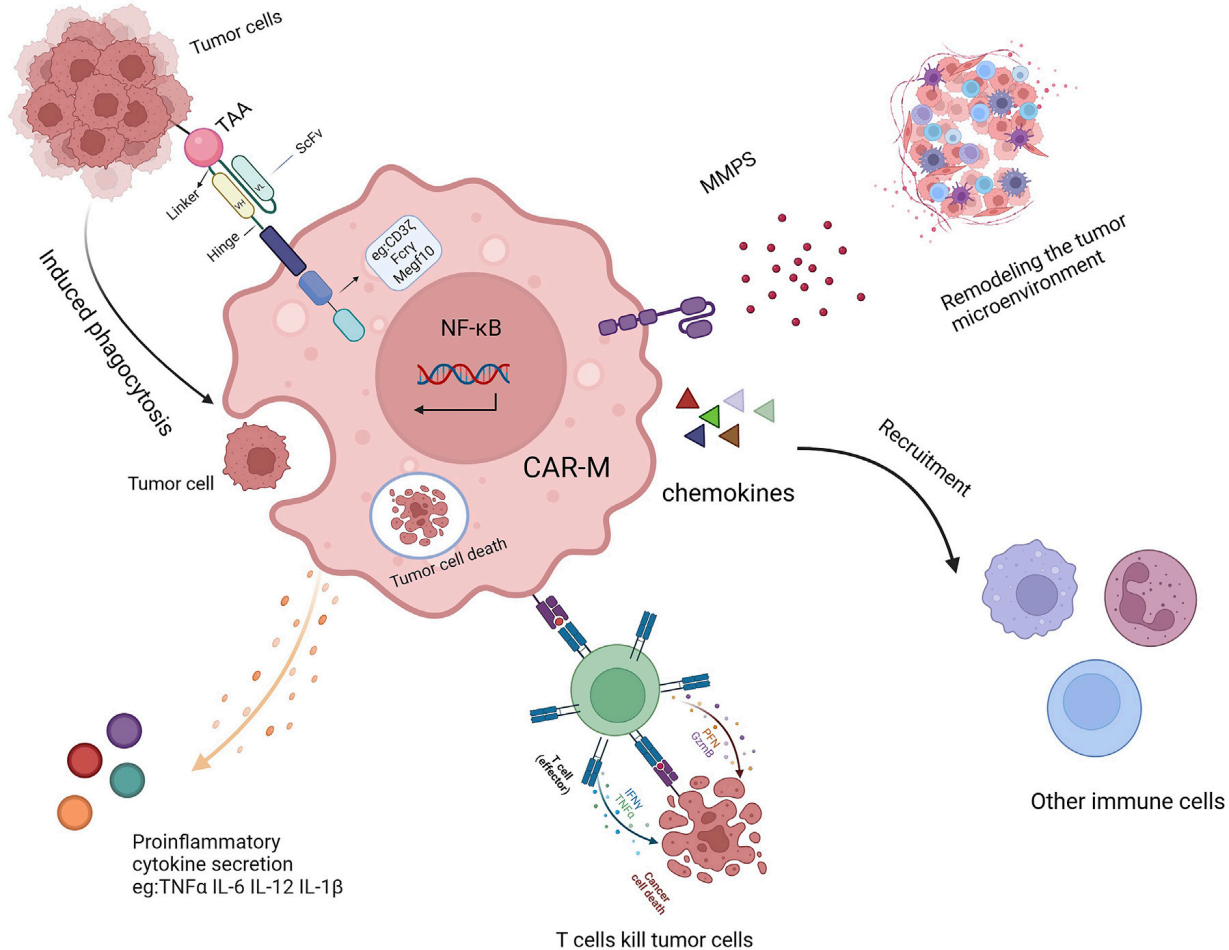
The mechanism of action of CAR-Ms includes TAA-induced phagocytosis, secretion of proinflammatory cytokines, activation of T cells, remodeling of the TME, and recruitment of other immune cells.

Once introduced into the body, CAR-Ms can mediate phagocytosis through their chimeric antigen receptor, secrete various cytokines to combat the immunosuppressive microenvironment (Figure 2) (Sloas et al., 2021; Huo et al., 2023; Ji et al., 2024), and release matrix metalloproteinases to reshape the microenvironment’s structure (Yang et al., 2024; Villanueva, 2020; Kuznetsova et al., 2024; Li N. et al., 2024). As antigen-presenting cells, CAR-Ms can also enhance the cytotoxicity of T cells, thereby boosting immune efficacy (Shin et al., 2023; Hadiloo et al., 2023; Li SY. et al., 2023). Additionally, CAR-Ms can recruit the body’s own macrophages to further support immune functions and achieve therapeutic effects (Liang et al., 2023; Xu et al., 2022).

Macrophages exhibit significant heterogeneity and have the ability to swiftly modify their functions in response to signals from their local microenvironment. (Leung and Wong, 2021; Arora et al., 2021). They are generally categorized into two main types based on their phenotypes and functional roles: M1 macrophages, which are proinflammatory and classically activated, and M2 macrophages, which are anti-inflammatory and alternatively activated (Zheng et al., 2021). The polarization of macrophages into either the M1 or M2 subtype is a highly regulated process that involves various key signaling pathways, as well as transcriptional, epigenetic, and post-transcriptional regulatory mechanisms. M1 macrophages are induced by lipopolysaccharide (LPS) and interferon (IFN)-γ (Hu et al., 2023; Lewis et al., 2022). They secrete cytokines such as tumor necrosis factor (TNF)-α, interleukin (IL)-1, IL-6, IL-12, IL-23, and inducible nitric oxide synthase (iNOS) to promote a pro-inflammatory Th1 response (Italiani and Boraschi, 2014; Wang DR. et al., 2022; Anderson et al., 2021). Additionally, they secrete chemokines (CXCL9, CXCL10, CXCL11) to recruit Th1 cells to sites of inflammation (Patel et al., 2017). M2 macrophages, on the other hand, are induced by cytokines such as IL-4 and IL-13. They suppress inflammation by activating signal transducer and activator of transcription (STAT) six and secrete IL-10, arginase (ARG), and transforming growth factor (TGF)-β (Kadomoto et al., 2021; Li M. et al., 2023; Guo and Qian, 2024). Macrophages exhibit remarkable plasticity *in vivo* and play dual roles in various human diseases, contributing to both protective and pathogenic processes. Macrophages can be tailored for specific diseases by inducing them into targeted states of differentiation. (Murray et al., 2014).

### 2.2 Cell source of CAR-Ms

**FIGURE 3:**
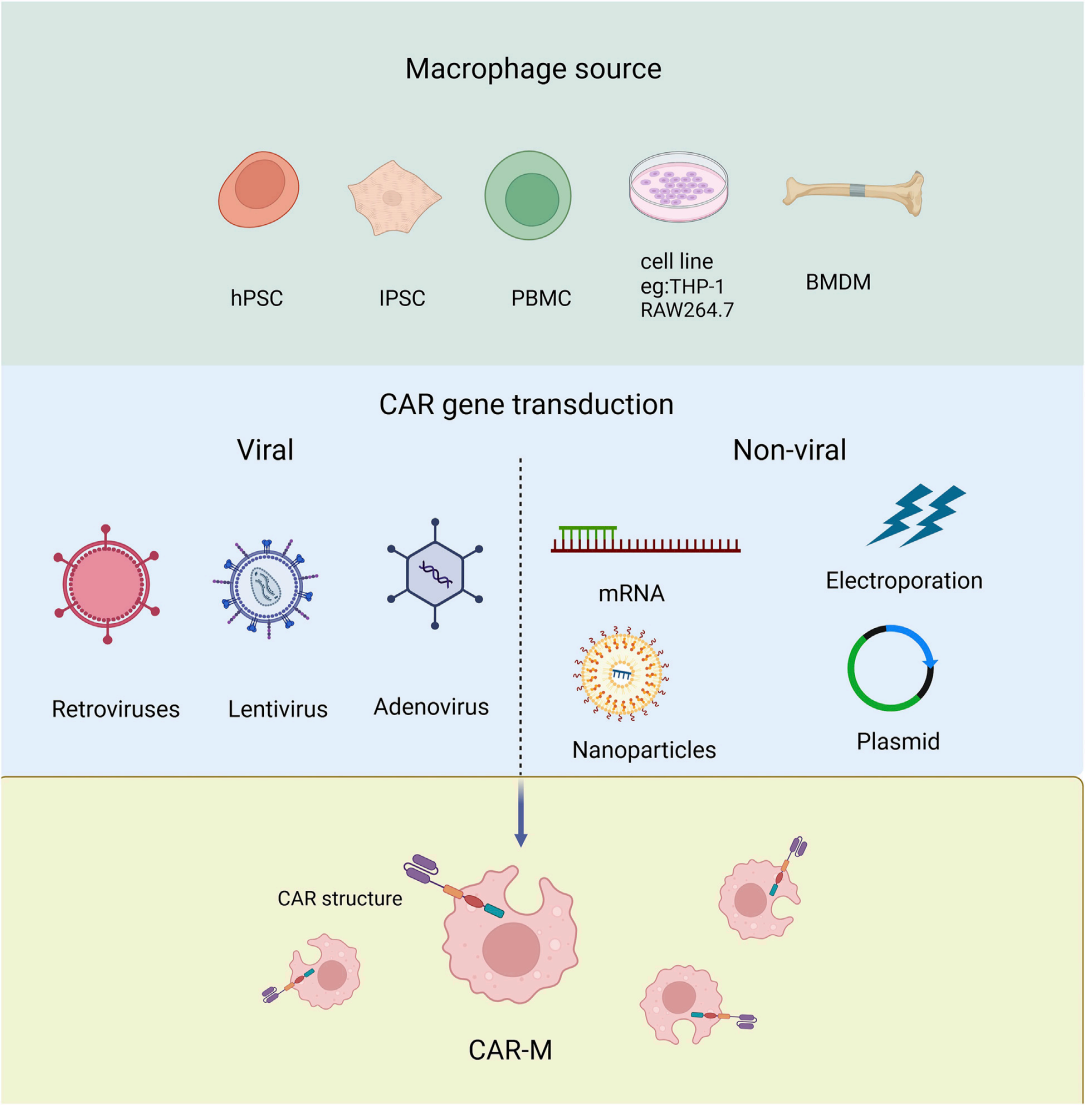
The source of macrophage used to produce CAR-Ms and the tools employed for transducing CAR genes.

Macrophages have been recognized as effector cells that destroy cancer cells due to their phagocytosis ability, so they have received much attention in the field of tumor immunotherapy (Alvey and Discher, 2017). Next, we will discuss the different origins of Human CAR-Ms which can be derived from a variety of ways and which can be modified to express car molecules (Figure 3).

Derived from cell line Human THP-1 cells can differentiate into CAR-macrophages after CAR engineering. The researchers attempted to evaluate the antigen-dependent phagocytotic ability of THP-1-derived macrophages, and the first generation of anti-CD19 CAR encoding CD3ζ intracellular segment was transduced to THP-1 cells. CD3ζ is homologous to the Fc co-gamma chain (FcεRI-γ), which is a specific classical signaling molecule for macrophages involved in antibody dependent cellular phagocytosis (ADCP). The results showed that CARs based on CD3ζ could enhance the anti-tumor phagocytosis activity of THP-1 macrophages (Sloas et al., 2021; Liu et al., 2022a; Su et al., 2022; Chocarro et al., 2022). Using RAW264.7 derived macrophages, the researchers designed CAR-Ms recognizing the tumor antigen HER2 (Morrissey et al., 2018).

Derived from monocytes Monocytes are widely distributed throughout the body, typically derived from peripheral blood or bone marrow (Biglari et al., 2006), and can be further differentiated into macrophages for the preparation of CAR-M. The peripheral blood monocytes (PBMC) with positive CD14 can be induced to macrophages by treating with granulocyte-macrophage colony-stimulating factor (GM-CSF) (Ball et al., 2022). While bone marrow-derived monocytes (BMMC) can be induced into mature macrophages by treating with macrophage colony-stimulating factor (M-CSF) (Dai et al., 2024). PBMCs are relatively easy to isolate and obtain in clinical, but have low differentiation efficiency than BMMC.

Pluripotent stem cells (PSCs) PSCs have the ability to differentiate into any type of cell in the body including macrophages. Embryoid bodies (EBs) represent the most prevalently employed approach for inducing PSCs to differentiate into macrophages. At present, the researchers have developed a highly efficient induction differentiation system, which can induce a single human PSC to produce about 6,000 macrophages within 2–3 weeks (Zhang et al., 2020; Netsrithong et al., 2023; Wang S. et al., 2022; Shen et al., 2024).

CAR-Ms sourced from different origins present distinct advantages and limitations. CAR-Ms derived from cell lines, such as THP-1, benefit from a stable genetic background, high operability, ease of culture, rapid proliferation, and convenient preservation. However, these immortalized macrophage cell lines are not suitable for clinical applications (Cousins et al., 2003; Mangan and Wahl, 1991; Mangan et al., 1991; Rogers et al., 2003; Gurunathan et al., 2021). On the other hand, CAR-Ms derived from monocytes retain their native form, offering better patient compatibility and specificity. Studies have shown that primary macrophages derived from PBMCs can produce higher levels of pro-inflammatory factors under M1 stimulation, but the availability of PBMCs is limited (Fuss et al., 2009; Schildberger et al., 2013). CAR-Ms derived from BMDMs are sourced from bone marrow and other tissues, which are relatively abundant, providing sufficient raw materials for CAR-M production. Additionally, PSCs offer a promising source for CAR-Ms, as they possess innate immune functions, including the expression and secretion of immune-related cytokines. The ease of amplifying PSCs, once the induction conditions and macrophage phenotypes are well established and characterized, makes them an attractive choice for producing CAR-Ms on a large scale (Zhang et al., 2020).

### 2.3 Preclinical and clinical studies of CAR-Ms

We conducted a search on PubMed and ClinicalTrials.gov using the keywords “chimeric antigen receptor macrophages” and summarized the preclinical and clinical studies on CAR-Ms in Table 1. The first report on CAR-Ms was published in 2017 by the team of Carl H. June and Saar Gill, pioneers in the field who initiated the first clinical trial of CAR-T cell therapy (Klichinsky et al., 2020). They explored the first-generation anti-CD19, anti-mesothelin, and anti-HER2 CARs in the THP-1 macrophage cell line and discovered that CAR-Ms could selectively phagocytose and eliminate cognate antigen-bearing tumor cells (Snyder and Gill, 2023). Subsequently, they introduced the anti-HER2 CAR into primary human macrophages, which demonstrated targeted phagocytosis and cytotoxicity against HER2-expressing ovarian and breast cancer cell lines (Klichinsky et al., 2020). In 2 mouse models of solid tumor xenotransplantation, a single infusion of human CAR-Ms was shown to reduce tumor burden and extend overall survival. Further studies in humanized mouse models revealed that CAR-Ms could induce a pro-inflammatory tumor microenvironment and enhance anti-tumor T cell activity. This groundbreaking work has since advanced to the clinical trial stage, as detailed in Table 1, representing the most advanced CAR-Ms cancer therapy to date.

**TABLE 1:** Preclinical and clinical trials of CAR-Ms.

Targeted antigen	Cell source	Gene delivery vector	Clinical/preclinical studies	Time
HER2	THP1 cell line	Adenoviral	preclinical	2017 (Snyder and Gill, 2023)
CD19, CD22	J774A.1cells	Lentivirus	preclinical	2018 (Morrissey et al., 2018)
HER2	Raw264.7 cells	Lentivirus	preclinical	2019 (Zhang et al., 2019)
CD19	iPSCs	Lentivirus	Preclinical	2020 (Zhang et al., 2020)
ALK	BMDMs	Nanocarrier	Preclinical	2021 (Kang et al., 2021)
CD133	THP-1 BMDMS RAW2647	Nanopore (NP)-hydrogel	Preclinical	2022 (Chen et al., 2022)
HER2, CD47	THP-1 cell line	Adenovirus	Preclinical	2023 (Chen et al., 2023)
HER2	BMDMs	Lentivirus	Preclinical	2023 (Huo et al., 2023)
GPC3 (Glypican-3)	Liver cancer site macrophages	Lipid nanoparticle	Preclinical	2023 (Yang et al., 2023b)
uPAR	BMDMs	Lentivirus	Preclinical	2024 (Dai et al., 2024)
HER2	Human monocytes	Adenoviral vector (Ad5f35)	NCT04660929	2020 (Klichinsky et al., 2020)
HER2	Autologous macrophages	Adenovirus	NCT06224738	2024 (Yang et al., 2024)

In 2018, Morrissey MA et al. created a new type of chimeric antigen receptor for phagocytosis, named CAR-P. By introducing CAR-P into macrophages, it was demonstrated that these modified cells could recognize and attack beads coated with proteins found on cancer cells. Additionally, they were able to limit the growth of live cancer cells *in vitro* by ‘biting’ and even ‘eating’ them (Morrissey et al., 2018).

In 2019, Zhang W et al. incorporated CD147 signaling molecule into the transmembrane region of HER2 CAR to reduce tumor collagen deposition and promote T-cell infiltration into tumors through effectively activating the expression of matrix metalloproteinases (MMPs) in macrophages after recognizing the antigen HER2. This work demonstrated that targeting the extracellular matrix (ECM) through engineered macrophages would be an effective treatment strategy for solid tumors (Zhang et al., 2019).

Zhang Jin’s team at Zhejiang University has made significant advancements in using induced pluripotent stem cells (iPSCs) to develop chimeric antigen receptor-expressing macrophages (CAR-iMac) for cancer immunotherapy. In November 2020, they were the first to report the successful application of CAR-iMac in both hematoma and solid tumor models in mice, demonstrating strong anti-cancer capabilities. In September 2023, they enhanced the efficacy of CAR-iMac against solid tumors by combining it with sialase and knocking down immune checkpoints Siglec-5 and Siglec-10 on macrophages (Wu et al., 2023). Additionally, they found that knocking out the Acod1 gene in CAR-iMacs led to a stronger pro-inflammatory response, improved phagocytosis, and enhanced tumor suppression in ovarian and pancreatic cancer models (Wang et al., 2023). Combining these modified CAR-iMacs with immune checkpoint inhibitors further improved tumor inhibition. Later that year, they developed a second-generation CAR-iMac with antigen-dependent polarization, enhancing cytotoxicity and antigen presentation, which offered a new method of tumor cell targeting called “cytoburial,” providing a solid theoretical foundation for CAR-iMac-based therapies in solid tumors (Lei et al., 2024).

In 2021, Kang’s team developed an innovative approach to cancer therapy by using nanoparticle carriers to transfect genes encoding CAR and IFN-γ into macrophages, creating MPEI/pCAR-IFN-γ. When injected directly into brain tumor-bearing mice (Neuro-2a), this treatment significantly extended the animals’ survival without causing notable changes in their body weight, indicating effective tumor inhibition with minimal side effects. This study marked a significant advance in cancer treatment by successfully converting macrophages into CAR-M1 type macrophages with potent anti-tumor activity, using a novel nanocomplex-mediated *in vivo* programming technique, offering new hope and challenges for cancer therapy (Kang et al., 2021).

In 2022, Professor Jiang Xinyi’s team from the School of Pharmacy at Shandong University, in collaboration with the School of Pharmacy at the University of Wisconsin-Madison, developed an injectable gene nanocarrier-hydrogel superstructure drug delivery system. In a mouse model of glioblastoma multiforme (GBM), this innovative system introduced a CAR gene targeting glioma stem cells (GSCs) into macrophage nuclei, resulting in the generation of GSC-specific CAR-Macrophages (CAR-Ms). These CAR-Ms were capable of specifically recognizing and phagocytizing GSCs, acting as antigen-presenting cells, and subsequently stimulating an adaptive anti-tumor immune response, ultimately forming immune memory (Chen et al., 2022).

In September 2023, the team of Tu Jiajie, Institute of Clinical Pharmacology, Anhui Medical University, constructed HER2-targeting CAR-Ms and CD47-targeting CAR-Ms, and co-cultured them with antigen-positive ovarian cancer cells *in vitro*. The results showed that HER2 CAR-Ms and CD47 CAR-Ms significantly inhibited tumorigenicity and tumor proliferation of ovarian cancer cell lines, confirming CAR-mediated phagocytosis of macrophages on ovarian cancer cells (Chen et al., 2023).

In 2023, Huo Y et al. conducted a study where macrophages were polarized *in vitro* using LPS combined with IFN-γ. The results showed a significant enhancement in the phagocytosis and killing ability of CAR-Macrophages (CAR-Ms) targeting cancer cells. Additionally, the expression of costimulatory molecules and proinflammatory cytokines increased markedly following polarization. By utilizing various *in vivo* isogenic tumor models, the researchers demonstrated that transfusions of these polarized M1 CAR-Ms effectively inhibited tumor progression, prolonged the survival of tumor-bearing mice, and enhanced overall cytotoxicity (Huo et al., 2023).

In 2023, by constructing an LNP that co-delivers CAR mRNA and mRNA encoding Siglec-G lacking ITIMs (Siglec-GΔITIMs), Yang Z et al. edited liver macrophages to generate phagocytic enhanced CAR macrophages in a mouse model of liver cancer. CAR-Ms generated by LNP and CD24-Siglec-G blockade can significantly improve the phagocytic function of liver macrophages, reduce tumor load and prolong survival time, providing an effective and flexible strategy for the treatment of HCC. This strategy avoids the complex procedure of making CAR-engineered cells *in vitro*, eliminates the potential side effects of systematic CAR-Ms transfusion, and has broad application prospects. Future studies will further evaluate the safety and efficacy of this strategy in clinical trials (Yang Z. et al., 2023).

In 2024, Wang Hua’s team at Anhui Medical University found that Mosaic antigen receptor-modified macrophages (CAR-Ms) can regulate the liver immune microenvironment to recruit and modify the activation of endogenous immune cells to drive the regression of fibrosis and produce specific anti-fibrotic T cell responses, providing the first preclinical evidence. Demonstrated that CAR-Ms targeting uPAR can enhance the immune response against liver fibrosis. Alleviating liver fibrosis and cirrhosis (Dai et al., 2024).

All the developments mentioned above are still in the pre-clinical experimental stage. For the clinical translation of CAR-Ms, careful attention must be given to their safety and efficacy. Although animal experiments have provided promising results, the safety and effectiveness of CAR-Ms in humans require further investigation (Chen et al., 2021). Notably, two CAR-Ms therapies have been registered with clinical trial numbers on the ClinicalTrials.gov website. (Tie et al., 2022; Schepisi et al., 2023). CT-0508 is an autologous cell therapy product consisting of pro-inflammatory macrophages derived from peripheral blood monocytes, developed by Carisma Therapeutics. It is the first CAR-M therapy to enter clinical trials, specifically a Phase I trial (NCT04660929) targeting HER2-overexpressing tumors. The HER2-directed CAR-Ms demonstrated a favorable safety profile and early signs of antitumor activity in patients with various solid tumors. Out of 14 patients treated, 28.6% achieved stable disease, all of whom had high HER2 expression (HER2+). Patients with lower HER2 expression (HER2-) experienced disease progression. No dose-limiting toxicities were reported, nor were severe cases of cytokine release syndrome (CRS) or neurotoxicity. Serious adverse events related to treatment were limited to grade 2 CRS and infusion reactions, which were manageable (Klichinsky et al., 2020; Li N. et al., 2024). Another early Phase I clinical trial (NCT06224738) was registered in March 2024 to evaluate human HER2-CAR-Ms therapy for HER2-positive advanced gastric cancer with peritoneal metastases. However, patient enrollment for the study has not yet been completed. (Yang et al., 2024).

Additionally, a Phase 2 randomized controlled clinical trial (ISRCTN 10368050) from the United Kingdom. Medicines and Healthcare products Regulatory Agency (MHRA) has evaluated the safety and feasibility of autologous macrophage from peripheral blood monocytes in patients with cirrhosis, providing valuable information and a theoretical foundation for subsequent efficacy studies (Brennan et al., 2021). This clinical trial evaluated the efficacy of RTX001 versus standard care in patients with compensated cirrhosis and a MELD score between 10 and 17. Patients were randomized into treatment groups receiving either one or three macrophage infusions, or standard care. The trial’s primary outcome was the change in MELD score over 90 days, with secondary outcomes including adverse events, fibrosis markers, and quality of life measures. Results showed a ΔΔMELD score of −0.87 in the treated group compared to the control group, approaching statistical significance (*p* = 0.06). The treated group also had fewer severe liver-related adverse events and deaths within 360 days of follow-up. However, there were no significant differences in non-invasive fibrosis markers or quality of life. The study concluded that macrophage therapy is safe and shows therapeutic potential in patients with cirrhosis, warranting further research (Brennan et al., 2021).

In conclusion, while the advancements in CAR-Ms therapy have shown significant promise in pre-clinical stages, translating these findings into clinical applications requires careful consideration of safety and efficacy in humans. The progression of CAR-Ms therapies into clinical trials, as evidenced by the FDA’s approval of candidates like CT-0508, marks a critical milestone in the field. These early-stage clinical trials have demonstrated encouraging results, particularly in targeting solid tumors, and have laid the groundwork for future exploration of CAR-Ms therapies. However, the continued development of CAR-Ms will necessitate further research to validate these therapies’ effectiveness and safety in diverse patient populations, ensuring that they can be reliably integrated into clinical practice.

## 3 CAR-M’s strengths, challenges and future direction

### 3.1 CAR-M’s strengths, and challenges

Compared with CAR-T and CAR-NK, CAR-Ms has its unique advantages as a new cellular immunotherapy (Table2), such as: 1). CAR-Ms can infiltrate tumor tissues in large quantities, reduce the proportion of TAMs, affect the phenotype of TAMs, and have a positive effect on tumor treatment (Su et al., 2022; Yoon et al., 2018). CAR-Ms possess the unique ability to reprogram the TME and sustain T cell infiltration. In a HER2-4T1 cold tumor model, the infusion of CAR-147 macrophages significantly inhibited tumor growth, leading to a fourfold increase in T cell infiltration compared to controls (Zhang et al., 2019). Similarly, Lei et al. demonstrated that combining anti-CD47 monoclonal antibodies with dual CD3ζ–TIR CAR macrophages resulted in superior tumor eradication in HEPG2 cells *in vivo* (Lei et al., 2024). In contrast, CAR-T therapies have shown limited success in solid tumor models, failing to produce satisfactory outcomes (Albelda, 2024). The effectiveness of CAR-M therapies against solid tumors is largely attributed to their ability to remodel the TME (Hadiloo et al., 2023). This remodeling is facilitated by the secretion of MMPs during antigen recognition, which degrade the ECM of the tumor. 2).In addition to phagocytosis of tumor cells, CAR-Ms can directly kill tumor cells expressing antigen by secreting cytokines (Keshavarz et al., 2022), promote antigen presentation and enhance T cell killing (Li W. et al., 2022; Uscanga-Palomeque et al., 2023). 3). Compared with CAR-T, CAR-Ms has limited time in circulation (Li SY. et al., 2023; Mercanti et al., 2023), moderate affinity of CAR-Ms can avoid unnecessary non-targeted toxicity, show little “off-target, non-tumor toxicity, and achieve better therapeutic effect (Su et al., 2022; Brudno and Kochenderfer, 2019). 4). CAR-Ms creates a pro-inflammatory environment in the tumor by secreting cytokines and chemokines and recruiting T cells and other white blood cells, reversing the inhibitory tumor microenvironment, and has also shown effective anti-tumor ability in animal experiments (Li W. et al., 2022). 5). CAR-Ms has the inherent tumor homing ability of myeloid cells, so it can enter solid tumors (Liu et al., 2022c).

**TABLE 2:** Pros and Cons of CAR-T Cells vs CAR-Ms.

Comparison range	CAR-T	CAR-Ms
Anti-tumor mechanism	Perforin-granzyme or death receptor-mediated cytotoxicity	Phagocytosis; antigen presentation; ECM degradation
Preparation time	2–4 weeks	From 1 month to 6 months
Infiltration into solid tumors	Harder	Good
Remodeling TME	—	Yes
Adverse reaction	Off-target toxicity; CRS Neurotoxicity	CRS observed; clinical data are insufficient

CAR-M therapy also faces some of the same challenges as CAR-T cell therapy, particularly regarding the time and cost of treatment (Pan et al., 2022). CAR-M therapy also faces some of the same challenges as CAR-T cell therapy, particularly regarding the time and cost of treatment (Kim and Bae, 2016). To address these issues, rapid CAR production processes, which enable CAR cell infusion within 24 h, and *in vivo* CAR induction strategies are being developed to shorten manufacturing timelines and reduce costs. Furthermore, Macrophages do not proliferate, and patients receive a limited number of macrophages, either *in vitro* or *in vivo*, which may affect the effectiveness of treatment. The ability of macrophage to remodel the TME and continuously stimulate T cell proliferation makes them a promising target for further enhancement. Strengthening these capabilities could significantly improve the effectiveness of CAR-M therapies. Additionally, the safety and efficacy of CAR-Ms in humans have yet to be confirmed by clinical trials (van der Heide et al., 2019). At present, CAR-Ms is mostly transfected by virus, which may lead to insertion mutation. The underlying mechanism of CAR-Ms resistance remains to be studied (Roth et al., 2018). (Chen et al., 2023; Zhang et al., 2023). The complex immune microenvironment should also be considered when applying CAR-Ms therapy (Nakamura and Smyth, 2020; Kochneva et al., 2020). Therefore, rational selection of existing therapies in combination with CAR-Ms may have a synergistic effect against tumor response.

### 3.2 Future direction of CAR-Ms therapy

#### 3.2.1 Optimization of CAR-Ms function

Several areas warrant further exploration to enhance the efficacy of CAR-M therapy in the future. First, in terms of CAR molecule design, structural optimization—such as the integration of diverse domains or the introduction of tandem activation domains—could be explored to refine the architecture of CAR-Ms (Hadiloo et al., 2023). These modifications may improve the phagocytic efficacy of CAR-Ms against cancer cells. Additionally, genetic engineering approaches to enable CAR-Ms to express anti-tumor cytokines or immunostimulatory molecules could enhance antigen presentation and further boost therapeutic efficacy (Moreno-Lanceta et al., 2023). Moreover, safety considerations can be addressed by designing multi-antigen logic gates or drug-sensitive modules to modify CAR-Ms, potentially improving their safety profile (Savanur et al., 2021). Non-viral delivery systems for CAR-encoding DNA or mRNA can also be employed to program macrophages into CAR-Ms (Sloas et al., 2021). Beyond direct cytotoxicity, CAR-Ms have demonstrated other anti-tumor capabilities, such as secreting MMPs to degrade the extracellular matrix and inducing epitope spreading (Kang et al., 2021; Chen et al., 2022; Komohara et al., 2016; Paasch et al., 2022).

#### 3.2.2 The future clinical applications of CAR-Ms on more diseases beyond cancers

**FIGURE 4:**
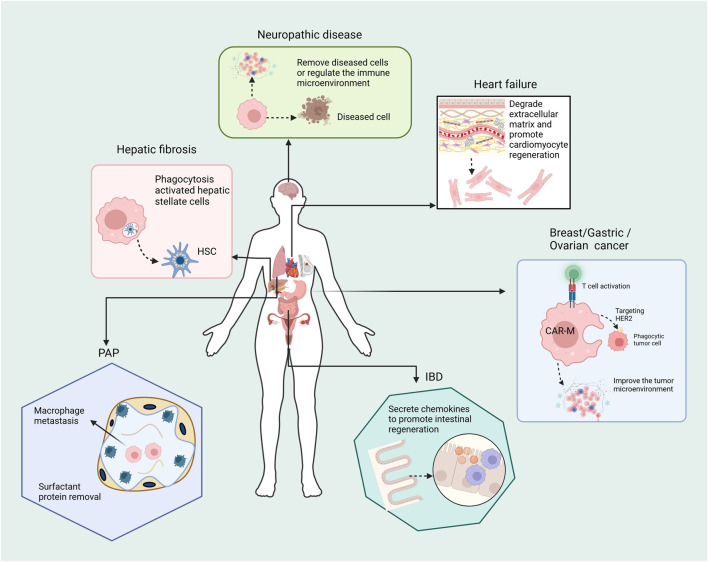
The future clinical applications of CAR-Ms extend to more diseases beyond cancers. Clear accumulated neurotoxic materials; Reduce extracellular matrix (ECM) contents around the heart injury site; Resolve the pathology of pulmonary alveolar proteinosis (PAP); Coordinate the regeneration of damaged intestinal epithelial cells; Resolve fibrosis and promote hepatocyte regeneration.

Macrophages possess remarkable regenerative capacity, directly aiding tissue reconstruction in various injured organs, including the intestines, skin, liver, heart, kidneys, and lungs (Figure 4). Their phagocytic ability can be harnessed to clear cancer cells, neurodegenerative debris, and infectious agents (Na et al., 2023). For instance, the infusion of CAR-Ms is expected to efficiently clear accumulated neurotoxic materials in genetic brain diseases (Shibuya et al., 2022). Additionally, specific subtypes of CAR-Ms may reduce ECM content around heart injury sites, promoting regeneration (Henry et al., 2014; Podaru et al., 2019). CAR-Ms can also secrete CXCL1 and WNT, which coordinate the regeneration of damaged intestinal epithelial cells, offering a potential treatment for inflammatory bowel disease (IBD) (Na et al., 2021). Furthermore, CAR-Ms can help resolve the pathology of pulmonary alveolar proteinosis (PAP) by clearing excessive surfactant (Suzuki et al., 2014).

As potent pro-inflammatory regulators, CAR-M therapies are emerging as a promising area of research for infectious and autoimmune diseases. Macrophages play a crucial role in inflammatory responses, and CAR-Ms have the potential to modulate the inflammatory microenvironment by expressing anti-inflammatory factors or cytokine receptors, thereby controlling excessive inflammation (Zouali, 2024). For example, Perez Amill et al. highlighted the broad range of potential applications of CAR-Ms in targeted antigen recognition, with promising implications for autoimmune diseases (Perez-Amill et al., 2021).

#### 3.2.3 CAR-Ms therapy in combination with other immunotherapeutic strategies

Although CAR-Ms have been proven effective in inhibiting tumors, the complexity of the tumor microenvironment and the challenges of cancer treatment have led researchers to explore combining CAR-Ms with other immunotherapies as a more comprehensive approach (Chen et al., 2021; Boshuizen and Peeper, 2020). For instance, blocking the CD47-SIRPα axis—the “do not eat me” signal—can enhance the phagocytic ability of CAR-Ms against cancer cells. Additionally, in patients with a high tumor burden, combining CAR-Ms with CAR-T cell therapy has emerged as a potential strategy. Since CAR-Ms are capable of suppressing cytokine release, their use alongside CAR-T therapy could reduce the risk of CRS and neurotoxicity, which are common complications associated with CAR-T treatments (Giavridis et al., 2018) (Shen et al., 2024). Liu et al. further investigated the synergistic potential of CAR-M and CAR-T cells in targeting tumor cells (Huang et al., 2024). They found that CAR-Ms and CAR-T cells demonstrated synergistic cytotoxicity against tumor cells *in vitro*. Inflammatory cytokines secreted by CAR-T cells induced M1 macrophage polarization, while increasing the expression of co-stimulatory ligands (CD86 and CD80) on CAR-Ms enhanced their cytotoxic effects. This study, for the first time, demonstrated the cooperative interaction between CAR-Ms and CAR-T cells in killing tumor cells, providing a novel avenue for combined immunotherapy approaches.

## 4 Prospects of CAR-Ms therapy

Any new invention must go through a long and arduous process from basic experiment to clinical application. Although there are still many difficulties to be overcome ahead, the future of CAR-Ms therapy holds significant promise for advancing medical treatments and addressing various diseases beyond its current applications. Real-world evidence from clinical practice will further inform best practices, optimize treatment protocols, and identify potential areas for improvement. Ongoing and future clinical trials will provide valuable insights into the safety, efficacy, and long-term effects of CAR-Ms therapy.
